# Comprehensive analysis of plasma cell heterogeneity and immune interactions in multiple myeloma

**DOI:** 10.3389/fimmu.2025.1549742

**Published:** 2025-04-22

**Authors:** Shuang Qu, Zhihai Zheng, Xiaoling Guo, Jiaqi Mei, Sicong Jiang, Biyun Chen

**Affiliations:** ^1^ Department of Hematology, Shengli Clinical Medical College of Fujian Medical University, Fuzhou University Affiliated Provincial Hospital, Fuzhou, China; ^2^ Translational Medicine Centre, the First Affiliated Hospital, Sun Yat-sen University, Guangzhou, China; ^3^ Department of Hematology, The Second Affiliated Hospital, Jiangxi Medical College, Nanchang University, Nanchang, China

**Keywords:** multiple myeloma, plasma cells, single-cell sequencing, weighted gene coexpression network analysis, tumor microenvironment

## Abstract

This study focused on the role of plasma cells in multiple myeloma (MM) and the associated potential mechanisms. Transcriptomic data of MM and various gene sets from several public databases were downloaded for subsequent analyses. Through single-cell sequencing, 10 major cell types were identified and annotated. The differential gene expression and pathway enrichment between different plasma cell subtypes as well as cell communication analysis, transcriptional regulation analysis, and enrichment analysis in conjunction with the malignant subpopulation were performed. Next, the samples were clustered into two groups by applying non-negative matrix factorization (NMF). Additional analysis revealed notable disparities in survival between the two clusters, correlation with genes involved in classical metabolic pathways and pathway dysregulation, thus confirming the stability and validity of the clustering. Subsequently, Weighted Gene Co-expression Network Analysis was performed and hub genes from the modules most strongly associated with the clustering groups were extracted. We then constructed a prognostic prediction model using Least Absolute Shrinkage and Selection Operator and multiCox regression analysis. The predictive accuracy of the model was evaluated and robustness were confirmed in a separate validation cohort. The gene and pathway dysregulation for the two risk groups was analyzed. Ultimately, an investigation was conducted into the association between the risk model and various immunological features, in terms of antitumor immunotherapy, the tumor microenvironment, and immune checkpoints. This study provides an in-depth investigation into the potential mechanisms underlying MM development and offers new directions to improve therapeutic approaches and enhance patient outcomes.

## 1 Introduction

Multiple myeloma (MM) is a malignant hematologic cancer accounting for 1% of all cancers (1) and 10% of all hematologic cancers (1, 2), making it the second most common hematologic malignancy worldwide (3). The incidence and mortality rates of MM vary region-wise, with age-standardized incidence rates being relatively low in regions, including Asia and Oceania, although the global trend shows an increasing incidence year by year (4). The average annual cost per treatment cycle for MM patients in Australia, the country with the highest incidence rate, is approximately $25,000 (5). For most individuals, MM remains an incurable (6), making it crucial to identify its underlying causes. Several risk factors have been identified, such as advanced age, male gender, and African American ethnicity (7). Monoclonal gammopathy of undetermined significance (MGUS) is also associated with enhanced risk of developing MM or related malignancies (8). However, many of these risk factors are uncontrollable. Recent studies suggest a potential correlation of body mass index with MM risk in adults (9), potentially representing the only modifiable risk factor for the disease. From an etiological perspective, studies suggest potentially major role of skeletal diseases in the onset of MM (1). Most cases of MM arise from asymptomatic precursor conditions, such as MGUS (1). The annual rate of transformation from MGUS to MM or other related malignancies is approximately 1% (8). The precursor stage known as smoldering multiple myeloma (SMM) can be clinically identified, exhibiting a tenfold higher conversion rate to MM within the initial 5 years post-diagnosis compared to that of MGUS (10). While MGUS and SMM are clinically asymptomatic, both conditions may increase the risks of venous thromboembolism, infections, osteoporosis, and fractures (11). In contrast, the clinical symptoms of MM include anemia, bone pain, fatigue, renal dysfunction, and infections (12). With advances in treatment, survival prognosis of patients with MM has greatly improved, as evidenced by the increase in the 5-year survival rate from 32% in 1996 to 54% in 2020 (13). Current treatments include the use of proteasome inhibitors such as bortezomib, immunomodulatory agents such as lenalidomide, and monoclonal antibodies such as daratumumab. The combination treatment of dexamethasone, lenalidomide, and bortezomib with autologous stem cell transplantation (ASCT) and subsequent maintenance therapy has demonstrated good and complete remission rates, with some studies reporting rates as high as 33% (14). However, the sad reality is that the greater number of patients with MM will eventually experience relapse. CAR T-cell therapy, bispecific T-cell engagers (BiTEs), and BCMA-targeted antibody-drug conjugates have demonstrated improved survival and remission rates in relapsed or refractory MM; however, both CAR T-cell therapy and BiTEs are associated with side effects such as cytokine release syndrome, immune effector cell-associated neurotoxicity syndrome, and other specific adverse reactions (15, 16). Therefore, the molecular mechanisms underlying MM development need to be studied for identifying key target genes, which have significant implications for the diagnosis, treatment, and prognostic evaluation of MM.

Common metabolic pathways include but are not limited to lipid metabolism, nucleotide metabolism, amino acid metabolism, and glucose metabolism. Cancer cells often engage in metabolic reprogramming to adapt to their specific growth characteristics and microenvironment. The Warburg effect is one of the most prominent metabolic alterations in cancer cells. Although the Warburg effect-induced aerobic glycolysis is inefficient, cancer cells meet their energy demands by upregulating glucose transporters (GLUT) and the enzymes required for glycolysis (17). Oncogenes, such as HIF-α, c-Myc, and Akt promote glycolysis through the upregulation of GLUT, while tumor suppressor genes like *p53* exert anti-cancer effects through the inhibition of glucose metabolism (18). In MM, cyclin D1 can regulate glycolysis and sustain the Warburg effect by binding to hexokinase 2 (HK2) on the outer mitochondrial membrane and controlling the transcription of the *HK2* gene (19). The reprogramming of lipid metabolism is a crucial element in the advancement of various malignancies (20). This phenomenon is primarily characterized by *de novo* lipogenesis (21), enhanced fatty acid uptake (22), and modified fatty acid oxidation pathways (23). The enzymes regulating these processes are often overexpressed in tumor cells. In MM, mature adipocytes induce resistance to chemotherapy in MM cells by secreting adipokines and activating autophagy (24). MM cells have been shown to promote tumor progression by reducing the secretion of adiponectin from bone marrow adipocytes (25). In reprogramming of amino acid metabolism, cancer cells show an increased dependence on glutamine, which enters the tricarboxylic acid cycle via glutamate and α-ketoglutarate to generate energy, making it a key metabolic pathway for cancer cells (26). MM cells exhibit glutamine dependency, and the inhibition of the glutamine transporter (ASCT2) markedly diminishes glutamine uptake, thereby suppressing the growth of MM cells (27). Consequently, targeting amino acid metabolism may be a promising therapeutic strategy (28). In case of low levels of amino acids, it may activate the eukaryotic initiation factor 2-alpha kinase, general control nonderepressible 2 (GCN2) (29), which leads to resistance to proteasome inhibitors (PIs). Combined use of ASCT2 inhibitors with PIs can enhance the sensitivity of MM cells to PIs by regulating glutamine levels (30). In nucleotide metabolism, enhanced synthesis of nucleoside triphosphates (NTPs) and deoxyribonucleoside triphosphates (dNTPs) is a hallmark of tumor cells, promoting cell proliferation, metastasis, immune evasion, and drug resistance. Nucleotide synthesis inhibitors continue to play a major role in the treatment of various cancers (31).

For subsequent analysis, we downloaded MM transcriptomic data and various gene sets from multiple public databases. We investigated the cellular heterogeneity in MM through single-cell sequencing. The single-cell data were classified into several cell subpopulations and annotated as 10 major cell types. Among these cell types, we focused particularly on the plasma cell subpopulation. We analyzed differences in gene expression and pathway enrichment for different plasma cell subtypes and performed cell communication analysis, transcriptional regulatory analysis, and enrichment analysis in conjunction with the malignant subpopulation. Subsequently, the non-negative matrix factorization (NMF) with the “brunet” method for clustering was employed, categorizing the samples into two clusters (C1 and C2). We then analyzed the differences in survival between the two groups, the correlation of genes related to classical metabolic pathways, and pathway dysregulation. The stability and robustness of the clustering were well-validated. We conducted a Weighted Gene Co-expression Network Analysis (WGCNA), extracting hub genes from the modules most closely associated with the clustering, and performed Gene Ontology (GO) analysis to identify the pathways enriched with these hub genes. We then constructed a prognostic prediction model using the Least Absolute Shrinkage and Selection Operator (LASSO) and multiCox regression analyses. The dataset was classified into high- and low-risk groups based on the median risk score, and the predictive performance and the model stability were validated in an independent validation cohort. Subsequently, gene and pathway dysregulation in the two risk groups were analyzed. Ultimately, we examined the correlation between the risk model and immunological features from several perspectives, in terms of antitumor immunotherapy, the tumor microenvironment (TME), and immune checkpoint pathways. This study provides an in-depth exploration of various potential mechanisms underlying the development of MM, and offers new avenues for improving MM treatment strategies and enhancing patient prognosis.

## 2 Materials and methods

### 2.1 Data acquisition and preprocessing

The comprehensive transcriptomic dataset for MM, along with the associated clinical information, were acquired from the public database Gene Expression Omnibus (GEO, https://www.ncbi.nlm.nih.gov/geo/) using the “GEOquery” R package. Two datasets, GSE136324 (n=867) and GSE136337 (n=426), were downloaded; all samples having a survival time ≤ 0 days were excluded. Additionally, single-cell RNA sequencing (scRNA-seq) data for MM, including the datasets GSE117156 and GSE164551, were obtained from the public Tumor Immune Single-cell Hub 2 (TISCH2, http://tisch.comp-genomics.org/home/) database. In cases with a gene had multiple entries in the expression matrix, the data were averaged across the multiple rows. Furthermore, we employed the “msigdbr” R package to retrieve lists of genes related to nucleotide metabolism, lipid metabolism, amino acid metabolism, and glycolysis-related genes from the Molecular Signatures Database (MSigDB, https://www.gsea-msigdb.org/gsea/msigdb/index.jsp). All publicly available open-source databases employed in this study are freely accessible without the need for supplementary ethical approval. The data collection as well as subsequent analyses were carried out in strict adherence to the applicable regulatory guidelines.

### 2.2 Single-cell sequencing analysis

Using the “Single-Cell Pipeline” (SCP, https://github.com/zhanghao-njmu/SCP) package and the “Seurat” package, we analyzed the single-cell sequencing datasets GSE117156 and GSE164551. The accuracy and reliability of subsequent analyses were ensured by implementing comprehensive quality control measures alongside data preprocessing. The criteria for quality control were: nFeature_RNA < 900 and percent.mt < 25. Batch effects across multiple samples were corrected through the “harmony” R package. Next, uniform Manifold Approximation and Projection (UMAP) was employed for reducing the dimensionality of the single-cell data (resolution = 0.6), allowing us to identify multiple cell subpopulations. The subpopulations were annotated utilizing information obtained from the TISCH database. A UMAP plot was generated to visualize the distribution of different cell clusters and their major cell types. We also employed a heatmap to display the expression patterns of top-specific markers in several major cell types. For further analysis, we subsequently focused on the plasma cell cluster. Dimensionality reduction and clustering of plasma cells were performed through t-Distributed Stochastic Neighbor Embedding (t-SNE), and the results were visualized accordingly. Differential expression analysis on the plasma cell subpopulations was performed and identified through t-SNE, utilizing the RunDEtest function (fc.threshold = 1, only.pos = FALSE). The differentially expressed genes (DEGs) were visualized using a volcano plot, and the top 10 genes exhibiting the most significant changes in expression levels were highlighted. Furthermore, GO enrichment analysis of the DEGs was conducted through the application of the RunEnrichment function (db = “GO_BP”, species = “Homo_sapiens”, DE_threshold = “avg_log_2_FC > log_2_(1.5) & p_val_adj < 0.05”). The top six enriched pathways were presented in a bar chart.

### 2.3 Cell communication, transcriptional regulation, and functional analysis based on plasma cells

We first utilized the CellChat and NicheNet methods to analyze the intercellular interactions between plasma cell subpopulations and malignant cell clusters and visualized the results. We hypothesized that a greater number and stronger intensity of communications (indicated by thicker lines) suggest a closer relationship, and focused on our subsequent analyses of plasma subpopulations more tightly associated with malignant cells. Subsequently, receptor-ligand interaction intensities between plasma cell subpopulations and malignant cells were presented through bubble plot visualizations, with statistical significance set at a p-value < 0.01. Subsequently, we employed the “SCENIC” tool to construct gene regulatory networks and evaluated the differential activity of 29 regulators across different plasma cell subpopulations and malignant cell clusters, visualized using heatmaps. We combined the results from the cell communication analysis, and visualized the specific scores of regulators in plasma_0 using scatter plots, annotating the top four most prominent regulators. The enrichment of hallmark pathways was performed using the Gene Set Variation Analysis of the plasma cell subpopulations and malignant cell clusters, and the results were visualized using heatmaps. Additionally, the expression levels of functional genes across various cell populations were presented using heatmaps, and subsequently, differential expression analysis was performed between the plasma_0 and plasma_1 subpopulations. The expression of genes with | log_2_(fold change plasma_0 vs. plasma_1) | > 1 were considered upregulated in plasma_0, while the reverse indicated upregulation in plasma_1. Dysregulated genes were presented in a volcano plot.

### 2.4 Non-negative matrix factorization clustering and survival analysis post-clustering

Next, the top four regulons and their corresponding targets as signatures were selected. Hierarchical clustering on GSE136324 was performed using the NMF method with the “brunet” algorithm. The number of clusters (K) varied between 2 and 10 to determine the optimal fit. We evaluated the clustering results based on seven criteria, including phenotype correlation coefficient, residuals, dispersion, residual sum of squares (RSS), explained variance, silhouette coefficient, and sparsity. The variation between these indicators was demonstrated by visualizing using line plots. The specific criteria for determining the optimal K value were as follows: (1) maximizing the phenotype correlation coefficient to enhance consistency between the clustering results and the original data, (2) optimizing dispersion and silhouette coefficient to improve the distinguishability and quality of the clusters, (3) selecting the last K value before significant reduction in dispersion and RSS to ensure model fitting, and (4) focusing on significant increases in explained variance while balancing sparsity to ensure the interpretability of the clustering results. According to the optimal K value, GSE136324 was divided into clusters C1 and C2, and survival analysis was performed on both clusters. The survival analysis was visualized utilizing the “ggsurvplot” function, a feature provided by the “survminer” package within the R programming environment.

### 2.5 Differential expression analysis of metabolism-related genes and gene set enrichment analysis

A differential expression analysis was performed on genes associated with key metabolic pathways (nucleotide metabolism, lipid metabolism, glycolysis, and amino acid metabolism) in clusters C1 and C2. The results were visualized using boxplots generated with the “ggplot2” package. Following this, a GSEA was performed on clusters C1 and C2 employing the “clusterProfiler” package, for contrasting the pathway enrichments between the two clusters. The results were further visualized using the “GseaVis” package. A positive Normalized Enrichment Score (NES) indicates significantly upregulated pathway in C2 relative to C1, while a negative NES indicates significant downregulation in C2.

### 2.6 Weighted gene co-expression network analysis

Then, WGCNA was performed to identify the appropriate soft threshold for constructing a scale-free network. The optimal soft threshold (β) was determined according to the Scale Independence and Mean Connectivity. Subsequently, a co-expression network was constructed using the best soft threshold, and modules were visualized through a gene clustering dendrogram. Subsequently, the correlation between modules and clinical traits was computed and depicted through a heatmap visualization. Values with p < 0.05 were considered statistically significant; blue represented negative correlations, red indicated positive correlations, and darker colors reflected stronger associations. For modules with > 200 characteristic genes, GO enrichment analysis was performed. The top five enriched pathways for each module were visualized through bar charts. Then, the module most closely associated with the cluster for hub gene extraction was selected and the hub genes were identified through the examination of the correlation between Gene Significance (GS) and Module Membership, employing the selection criteria of GS > 0.4 and Module Membership > 0.6. The results were visualized through a scatter plot, and colored points represented hub genes. Ultimately, the identified hub genes were subjected to GO enrichment analysis, and the five top-most GO terms in Molecular Function (MF), Biological Process (BP), and Cellular Component (CC) were represented using bar chart visualizations.

### 2.7 Construction and validation of the prognostic model

LASSO regression analysis was performed using the GSE136324 dataset. Initially, the optimal regularization parameter λ was selected through the R package “glmnet.” The best fitting of the model was considered when the deviance, represented on the y-axis of the cross-validation curve, corresponding to the optimal log(λ) value was minimized. Based on this optimal λ, key genes with significant contributions to the LASSO regression equation were identified from the coefficient path distribution plot. Following this, we performed a multi-Cox proportional hazards regression analysis on the selected key genes to ascertain their respective coefficients, which were subsequently presented using a lollipop plot visualization. Subsequently, a predictive model was prepared, employing the chosen genes and their corresponding coefficients as fundamental determinants. Risk scores for each patient were determined; their subsequent categorization into high and low-risk groups was based on the median score. These groups were visualized using a dot plot. Survival and time-dependent Receiver Operating Characteristic (ROC) curves on the GSE136324 dataset were analyzed. These analyses compared prognostic disparities between the low-risk and high-risk groups, and the model’s accuracy in predicting outcomes was assessed. The model was subsequently re-applied to the GSE136337 validation dataset to further evaluate its predictive efficacy and stability.

### 2.8 Biological differences between high-risk and low-risk groups

A differential analysis between the high-risk and low-risk groups was performed utilizing the “limma” package, for identifying the top 20 dysregulated feature genes that differed between the two groups. Subsequently, a heatmap was employed to represent the expression profiles of these 20 genes across both the high- and low-risk groups. Following this, the expression levels of nine dysregulated immune checkpoints were analytically assessed. These were subsequently juxtaposed between the two groupings, and the differential expression patterns were also depicted through a heatmap visualization. Next, we conducted GSEA on the high-risk group using the “clusterProfiler” package. Gene sets were obtained from the Kyoto Encyclopedia of Genes and Genomes (KEGG) database, and the ridge plots for visualizing dysregulated KEGG pathways were generated using the “GseaVis” package. Furthermore, the Cancer Hallmark gene sets were acquired from the MSigDB database and used the “GseaVis” package to generate ridge plots for visualizing dysregulated Hallmark pathways. In these visualizations, greater intensity of blue color indicated a more statistically significant the pathway. Furthermore, when the value on the x-axis >0.0, indicated upregulated pathway, while values < 0.0 indicate downregulation.

### 2.9 Association between prognostic models and immunological features

The correlation between immune therapy pathways, anti-cancer immune cycle pathways, and risk scores were analyzed, and the results were visualized using correlation butterfly plots generated by “ggplot”. A significant negative correlation was indicated by p < 0.05 and Pearson’s r < -0.2, while p < 0.05 and Pearson’s r > 0.2 indicated a significant positive correlation. Subsequently, the TME analysis and quantification were performed using the “ESITMATE” algorithm from the “IOBR” package. This analysis yielded the ESTIMATE Score, Immune Score, and Stromal Score for the high-risk and the low-risk groups, respectively, and the results were visualized via box plots. Additionally, five drugs (Foretinib, JAK, Fludarabine, Erlotinib, Linsitinib) we selected for drug sensitivity analysis. The predicted half-maximal inhibitory concentration (IC50) values of these drugs were estimated and compared for the two risk groups, with the results were represented through box plot visualizations. Additionally, the CIBERSORT algorithm within the “IOBR” package was utilized to evaluate the correlation between risk scores and the extent of immune cell infiltration. The correlation of risk scores with six immune cell subpopulations were visualized using scatter plots. A p-value < 0.05 and R < 0 indicated a negative correlation; a larger absolute value of R indicated a stronger correlation. We further examined the correlation with respect to the 25 model genes and the infiltration levels of 22 distinct immune cell subpopulations across 10 immune cell types through Pearson’s correlation coefficients, and the results were visualized in the form of a correlation heatmap. In the concluding phase, a comprehensive examination was conducted to elucidate the relationship of the 25 model genes with 57 immune checkpoints, and the findings were subsequently depicted through a correlation heatmap.

### 2.10 Statistical assessment

Survival analysis was performed using the Kaplan-Meier (KM) method, and the log-rank test was used to compare the survival curves of high- and low-risk groups. The area under the ROC curve (AUC) was calculated, and an AUC > 0.55 was considered to indicate good test performance. A p-value < 0.05 was considered statistically significant in all analyses. Data were statistically analyzed using R software (version 4.3.1), and p < 0.05 was regarded as statistically significant. (*: p < 0.05; **: p < 0.01; ***: p < 0.001; ****: p < 0.0001).

## 3 Results

### 3.1 Data acquisition and preprocessing

**Figure 1:**
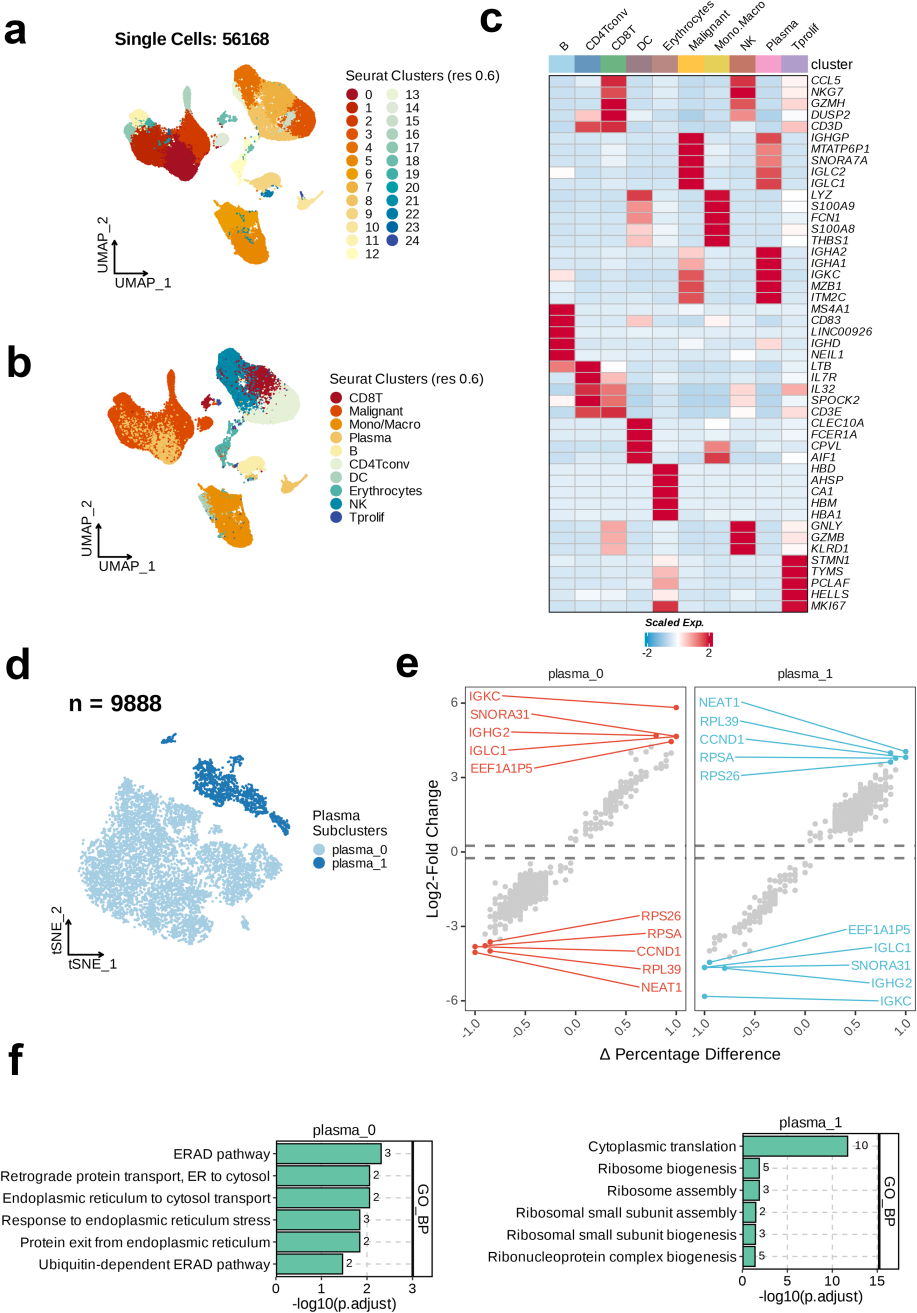
Single-cell RNA sequencing (scRNA-seq) analysis unravels the heterogeneity of plasma cells in MM. **(a)** The integrated scRNA-seq dataset revealed 25 clusters were identified in. **(b)** 10 major cell types were annotated. **(c)** Heatmap showing the top cell-type specific markers. **(d)** uniform Manifold Approximation and Projection visualization of the 9888 of plasma cells. **(e)** Volcano plots of the top ten dysregulated genes of each plasma subtype. **(f)** Top five enriched GO_BP terms of each plasma cell subset.

After quality control, data cleaning, and batch-effect correction of the GSE117156 and GSE164551 single-cell datasets, 56,168 single cells were obtained. Subsequently, the processed single-cell data were subjected to dimensionality reduction and clustering via the implementation of UMAP, leading to the identification of 25 discrete cell clusters (**Figure 1a**). Based on the TISCH database annotations, these 56,168 single cells were categorized into 10 major cell types, including CD8 T cells, B cells, malignant cells, plasma cells, mono/macrophages, CD4 T conventional (CD4Tconv) cells, dendritic cells (DC), erythrocytes, natural killer (NK) cells, and proliferating T cells (Tprolif) (**Figure 1b**).

Next, analysis of the expression of 47 specific markers across the 10 cell subtypes revealed the most prominently expressed markers in the B cell subtype, including MS4A1, CD83, LINC00926, IGHD, and NEIL1. The key markers in the CD4Tconv subtype were LTB, IL7R, IL32, and SPOCK2. The significant markers for CD8 T cells were CCL5, NKG7, GZMH, DUSP2, and CD3D. The top markers in the DC subtype included CLEC10A, FCER1A, CPVL, and AIF1. The erythrocyte subtype exhibited high expression of HBD, AHSP, CA1, HBM, and HBA1. The most notable markers in malignant cells were IGHGP, MTATP6P1, SNORA7A, IGLC2, and IGLC1. The mono/macrophage subtype was characterized by the expression of LYZ, S100A9, FCN1, S100A8, and THBS1. NK cells primarily expressed CCL5, NKG7, GNLY, GZMB, and KLRD1. Plasma cells showed enhanced expression of IGHA2, IGHA1, IGKC, MZB1, and ITM2C. Finally, in the Tprolif subtype, high expression of STMN1, TYMS, PCLAF, HELLS, and MK167 was noted (**Figure 1c**). This study focused on the plasma cell subpopulation. Therefore, we selected the plasma cell subtype (n=9888) for t-SNE dimensionality reduction and clustering, and found two distinct plasma cell subclusters, plasma_0 and plasma_1. The majority of the plasma cell population consisted of plasma_0 cells (**Figure 1d**). We then calculated the changes in the gene expression level between the two subclusters and annotated the top 10 dysregulated genes. In plasma_0, the most significantly upregulated genes relative to plasma_1 were IGKC, SNORA31, IGHG2, IGLC1, and EEF1A1P5. In contrast, the genes most significantly downregulated in plasma_0 relative to plasma_1 were RPS26, RPSA, CCND1, RPL39, and NEAT1 (**Figure 1e**). In the final step, GO enrichment analysis on the two plasma-cell subclusters revealed the top six enriched pathways in the GO_BP category. As shown in the bar chart, the most significantly enriched pathway in plasma_0 was the Endoplasmic Reticulum-Associated Degradation pathway, whereas the most enriched pathway in plasma_1 was Cytoplasmic Translation (**Figure 1f**).

### 3.2 Cell communication, transcriptional regulation, and functional analysis based on plasma cells

**Figure 2:**
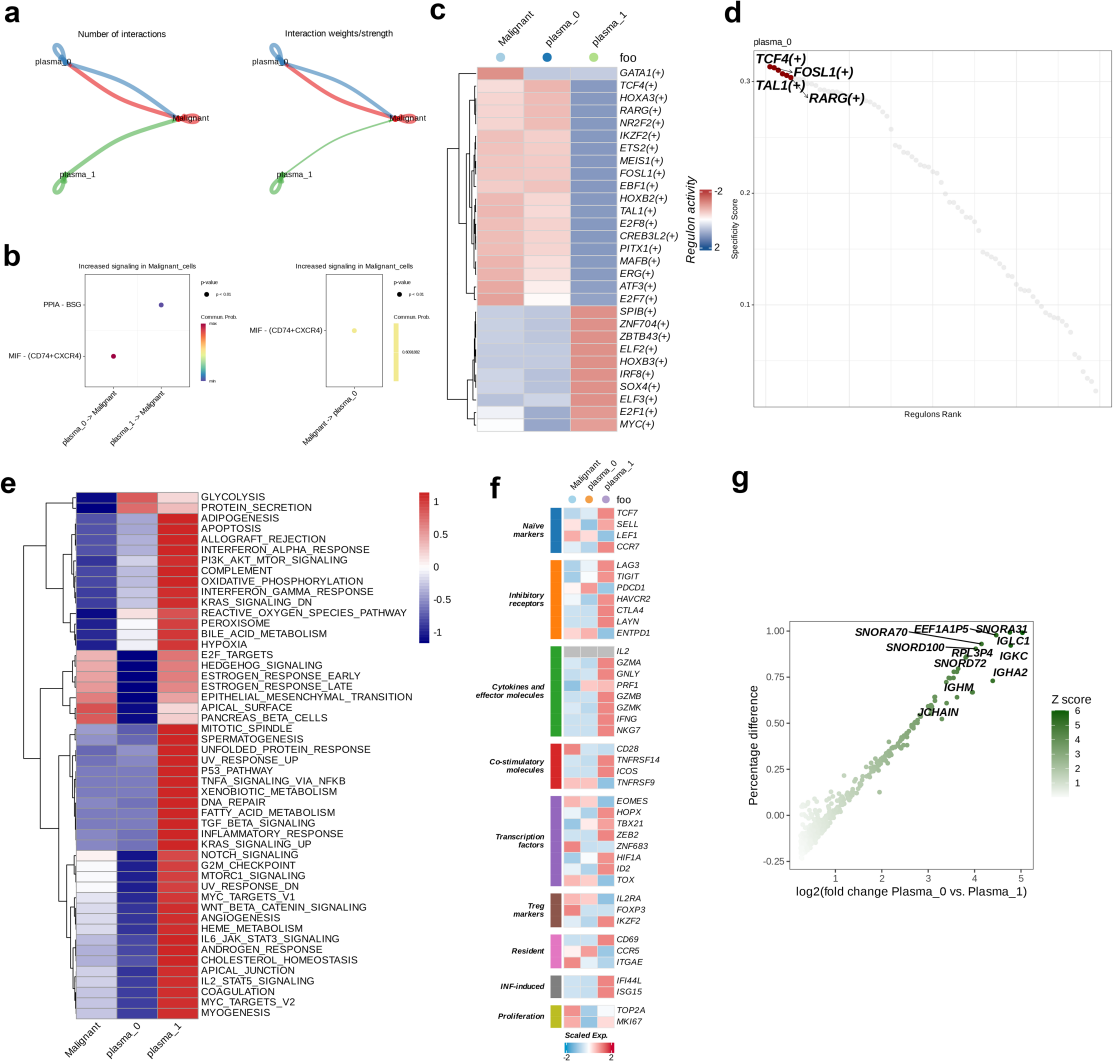
Intercellular communications between plasma and malignant cells. **(a)** The intercellular interactions between plasma and malignant cell subsets. **(b)** The ligand-receptor pairs between plasma and malignant cells. **(c)** Heatmap demonstrating the activity of each regulon in plasma and malignant cells. **(d)** The top four activated positive regulons of plasma subtype 0. **(e)** The enrichment properties of hallmarks of plasma and malignant cells. **(f)** Heatmap demonstrating the expression levels of functional genes of plasma and malignant cells. **(g)** Volcano plot showing the genes upregulated in plasma subtype 0.

Cell communication analysis on the two plasma cell subtypes and the malignant cell subtype identified through dimensionality reduction clustering revealed the number and strength of interactions between the malignant and plasma subgroups. The comparison presented in the figure reveals that plasma_0 exhibits more numbers of and stronger interactions with the malignant subtype, whereas the interaction strength from plasma_1 to malignant cells is weaker (**Figure 2a**). Therefore, for further exploration we selected plasma_0, showing closer interactions with malignant cells,. Next, we analyzed the signaling intensity of receptor-ligand interactions across different cell subtypes. PPIA-BSG exhibited lower signaling strength in the interaction between plasma_1 and malignant cells, whereas higher signaling intensity was observed in MIF-(CD74+CXCR4) in the interaction from plasma_0 to malignant cells. The signaling strength from malignant to plasma_0 was 0.609, suggesting a potential role of this interaction in the communication between malignant and plasma_0 cells (p < 0.01, **Figure 2b**). The activity of 29 regulatory factors across the different cell subtypes was then evaluated and the heatmap analysis revealed a similar trend of the activity of regulators in the plasma_0 and malignant cell populations, in contrast to plasma_1. Most regulatory factors showed upregulated activity in plasma_0 and malignant cells (**Figure 2c**). The specificity scores of regulatory factors in plasma_0 were calculated and ranked in descending order. The top six factors included TCF4 (+), FOSL1 (+), TAL1 (+), and RARG (+) (**Figure 2d**). Following this, GSVA on the three cell populations revealed that in the malignant cells, the expression of six hallmarks related to cell proliferation, migration, differentiation, and environmental adaptation, including E2F_TARGETS, HEDGEHOG_SIGNALING, ESTROGEN_RESPONSE_EARLY, ESTROGEN_RESPONSE_LATE, and APICAL_SURFACE, was more active. In contrast, plasma_0 cells showed upregulated activity in GLYCOLYSIS and PROTEIN_SECRETION; in contrast, the expression of other hallmarks was relatively low in both malignant and plasma_0 cells. In plasma_1, an opposite trend was noted in the expression of 50 hallmarks, with most of them being highly active in this subtype (**Figure 2e**). The eight immune-related cell pathways included naive markers, Treg markers, Resident, INF-induced pathways, Inhibitory receptors, Cytokines and effector molecules, Co-stimulatory molecules, and Transcription factors. Most functional genes showed more active expression in plasma_1, while an opposite trends was observed in in malignant and plasma_0 cells (**Figure 2f**). In the final step, a comparative analysis of differential gene expression was carried out to compare the gene profiles between plasma_0 and plasma_1. The volcano plot indicated that 11 genes, including SNORA31, EEF1A1P5, IGLC1, SNORA70, and SNORD100, were significantly upregulated in plasma_0, relative to plasma_1 (**Figure 2g**).

### 3.3 Non-negative matrix factorization clustering and post-clustering survival analysis

**Figure 3:**
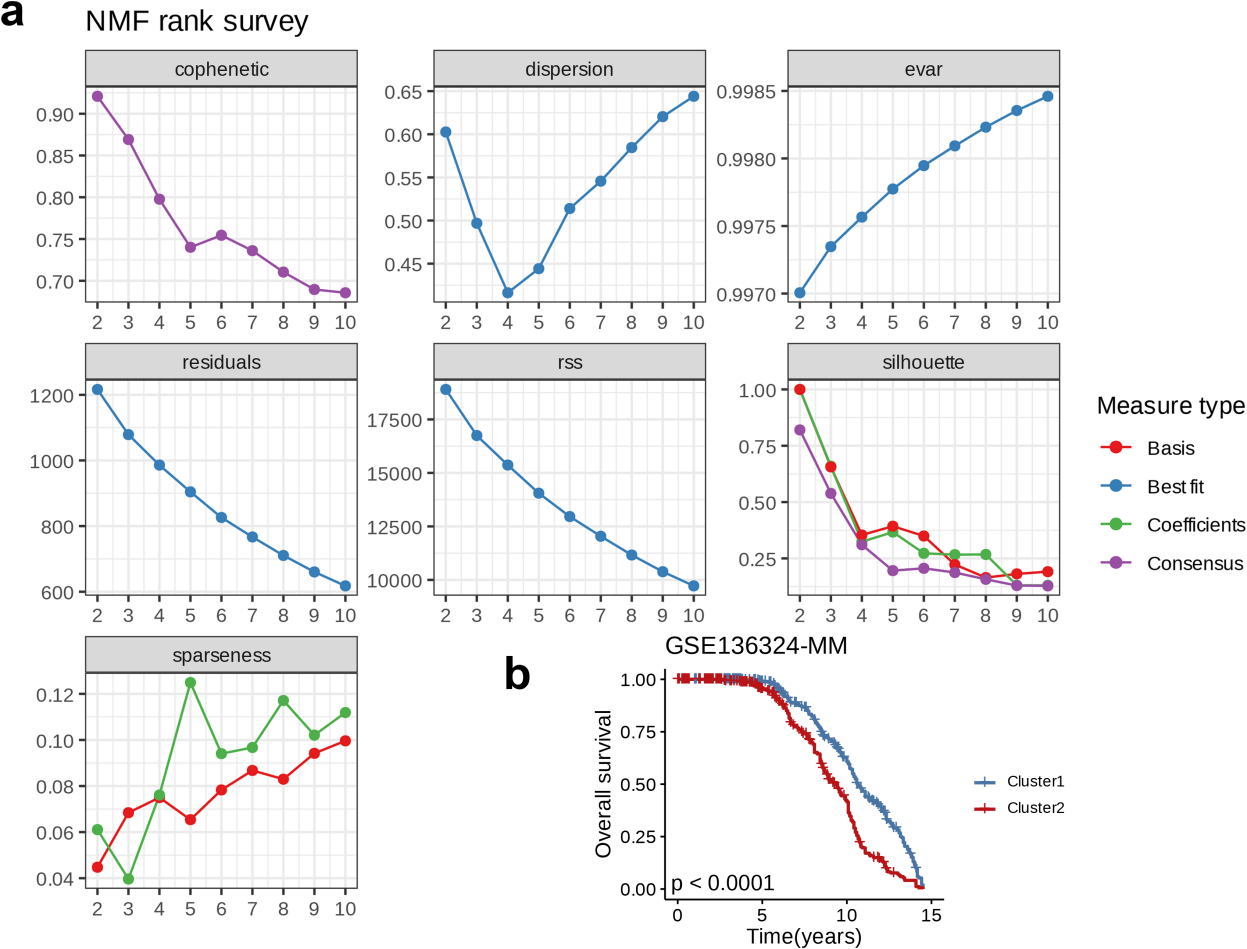
Nucleotide metabolism subclusters and prognosis in The Cancer Genome Atlas-Ovarian carcinoma. **(a)** Cophenetic distributions, residual sum of squares, and dispersion indices for ranks 2–10. **(b)** Overall Kaplan-Meier survival curves for both subclusters.

NMF on the GSE136324 dataset and a comprehensive analysis using seven indicators revealed phenotype correlation coefficient, residuals, dispersion, RSS, explained variance, silhouette coefficient, and sparsity. The optimal K-value was determined to be 2 (**Figure 3a**). Based on this, we classified the GSE136324 dataset into two clusters, C1 and C2, and conducted survival analysis on both clusters, and found that over time, the overall survival (OS) rate of cluster C2 was significantly lower than that of C1, and the two groups exhibited notable prognostic difference (p < 0.0001, **Figure 3b**).

### 3.4 Differential expression analysis of metabolism-related genes and gene set enrichment analysis

**Figure 4:**
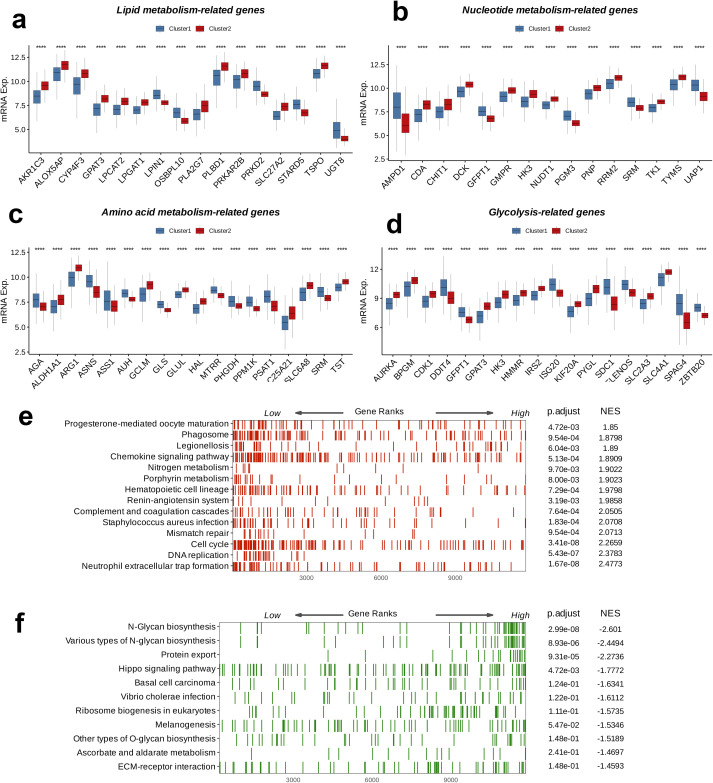
Crosstalk between nucleotide metabolism subclusters and key metabolic pathways. Differences between subclusters **(a)** in terms of lipid metabolism-related genes, **(b)** in terms of nucleotide metabolism-related genes, **(c)** in amino acid metabolism-related genes, **(d)** in glycolysis-related genes, and (B) in amino acid metabolism-related genes. Gene set enrichment analysis (GSEA) reveals **(e)** pathways upregulated in subtype C2 relative to C1 and **(f)** pathways downregulated in subtype C2 relative to C1. ****: p < 0.0001.

An analysis of DEGs related to metabolism was carried out to examine their expression patterns between C1 and C2. Among the 16 genes involved in lipid metabolism, 11 genes, including AKR1C3, ALOX5AP, CYP4F3, GPAT3, LPGAT1, PLA2G7, PLBD1, PRKAR2B, SLC27A2, and TSPO, were highly expressed in C2, whereas five genes, LPIN1, OSBPL10, PRKD2, STARD5, and UGT8, showed higher expression in C1 (p < 0.0001, **Figure 4a**). Among the 15 genes involved in nucleotide metabolism, 10 genes, including CDA, CHIT1, DCK, GMPR, HK3, NUDT1, PNP, RRM2, TK1, and TYMS, were highly expressed in C2, while five genes, AMPD1, GFPT1, PGM3, SRM, and UAP1, were more highly expressed in C1 (p < 0.0001, **Figure 4b**). For the 18 genes related to amino acid metabolism, eight genes, including ALDH1A1, ARG1, GCLM, GLUL, HAL, C25A21, SLC6A8, and TST, were highly expressed in C2, whereas 10 genes, including AGA, ASNS, ASS1, AUH, GLS, MTRR, PHGDH, PPM1K, PSAT1, and SRM, were more highly expressed in C1 (p < 0.0001, **Figure 4c**). Among the 18 glucose metabolism-associated genes, 11 genes, including AURKA, BPGM, CDK1, GPAT3, HK3, HMMR, IRS2, KIF20A, PYGL, SLC2A3, and SLC4A1, were highly expressed in C2, while seven genes, DDIT4, GFPT1, ISG20, SDC1, ELENOS, SPAG4, and ZBTB20, showed higher expression in C1 (p < 0.0001, **Figure 4d**). In summary, compared to C, the classical metabolic pathways were more active in C2. Further GSEA in both C1 and C2 indicated upregulation immune response-related pathways, such as Phagosome, Chemokine signaling pathway, and Neutrophil extracellular trap formation, as well as cell cycle-related processes, including Mismatch repair, Cell cycle, and DNA replication, in C2 relative to C1 (p < 0.05, NES > 0, **Figure 4e**). On the other hand, pathways related to protein synthesis, modification, and secretion, such as N-Glycan biosynthesis, various types of N-glycan biosynthesis, Protein export, Ribosome biogenesis in eukaryotes, and other types of O-glycan biosynthesis, along with cell signaling pathways such as the Hippo signaling pathway and ECM-receptor interaction, were downregulated in C2 relative to C1 (p < 0.05, NES < 0, **Figure 4f**).

### 3.5 Weighted gene co-expression network analysis

**Figure 5:**
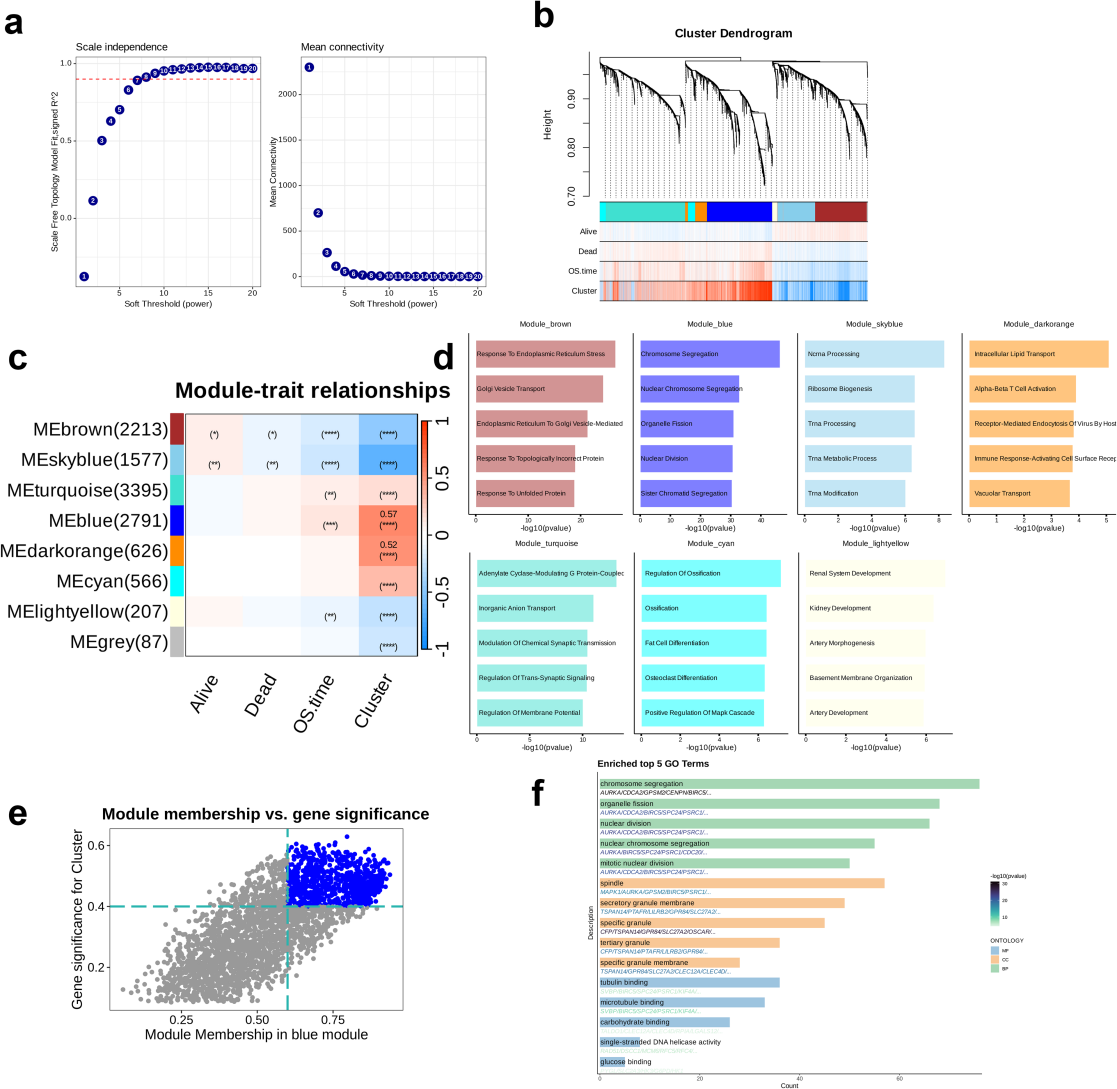
Identification oof key module related to clusters through Weighted Gene Co-expression Network Analysis (WGCNA). **(a)** Analysis of network topology for different soft-threshold power. The left panel showing the impact of soft-threshold power (power = 7) on the scale-free topology fit index; the right panel shows the impact of soft-threshold power on the mean connectivity. **(b)** Cluster dendrogram of the co-expression modules. Each color indicates a co-expression module. **(c)** Module-trait heatmap presenting the correlation between module eigengenes and clinical traits. **(d)** Top five enriched Gene Ontology terms of module genes in each module except for the grey module. **(e)** Correlation between module membership and gene significance is presented in the blue module. Colored dots were regarded as the hub genes of the corresponding module (MM > 0.6 & GS > 0.4). **(f)** Enrichment analysis of the hub genes.

WCGNA was performed by combining the scale-free topology fit index and the average connectivity plot, and the optimal soft threshold was identified as 7 (**Figure 5a**). A co-expression network was constructed based on the optimal soft threshold that we determined; the gene clustering dendrogram revealed a total of eight co-expression modules (**Figure 5b**). Subsequently, the correlations and significance between the modules and clinical features were analyzed to identify the strongest correlation between Module_blue and Cluster (p < 0.0001, R = 0.57, **Figure 5c**). Except for Module_grey, GO enrichment analysis was performed on the remaining seven modules, and the top five enriched pathways in each were identified. The most significantly enriched pathway in Module_brown was “Response to Endoplasmic Reticulum Stress,” while that in in Module_blue was “Chromosome Segregation”. In Module_skyblue, “Ncrna Processing” was the most prominent, whereas “Intracellular Lipid Transport” was most enriched in Module_darkorange. The most significantly enriched pathway in Module_turquoise was “Adenylate Cyclase-Modulating G Protein-Coupled,” followed by “Regulation of Ossification” in Module_cyan, and “Renal System Development” in Module_lightyellow. These pathways cover a range of critical biological processes, including cellular stress responses, cell cycle regulation, RNA processing, lipid metabolism, signal transduction, bone metabolism, and renal development (**Figure 5d**). Next, focusing on Module_blue, we analyzed the correlation between Module Membership and GS and identified the hub genes within the module (**Figure 5e**). The most significantly enriched GO_BP pathway was “Chromosome Segregation,” the most enriched GO_MF pathway was “Tubulin Binding,” and the most enriched GO_CC term was “Spindle.” These three pathways play essential roles in pivotal functions, including cell division, cytoskeletal organization, and genomic stability, all of which being crucial steps in the cell division process. Particularly, the enrichment of genes in the GO_BP pathway was relatively more significant (**Figure 5f**).

### 3.6 Development and confirmation of a prognostic model

**Figure 6:**
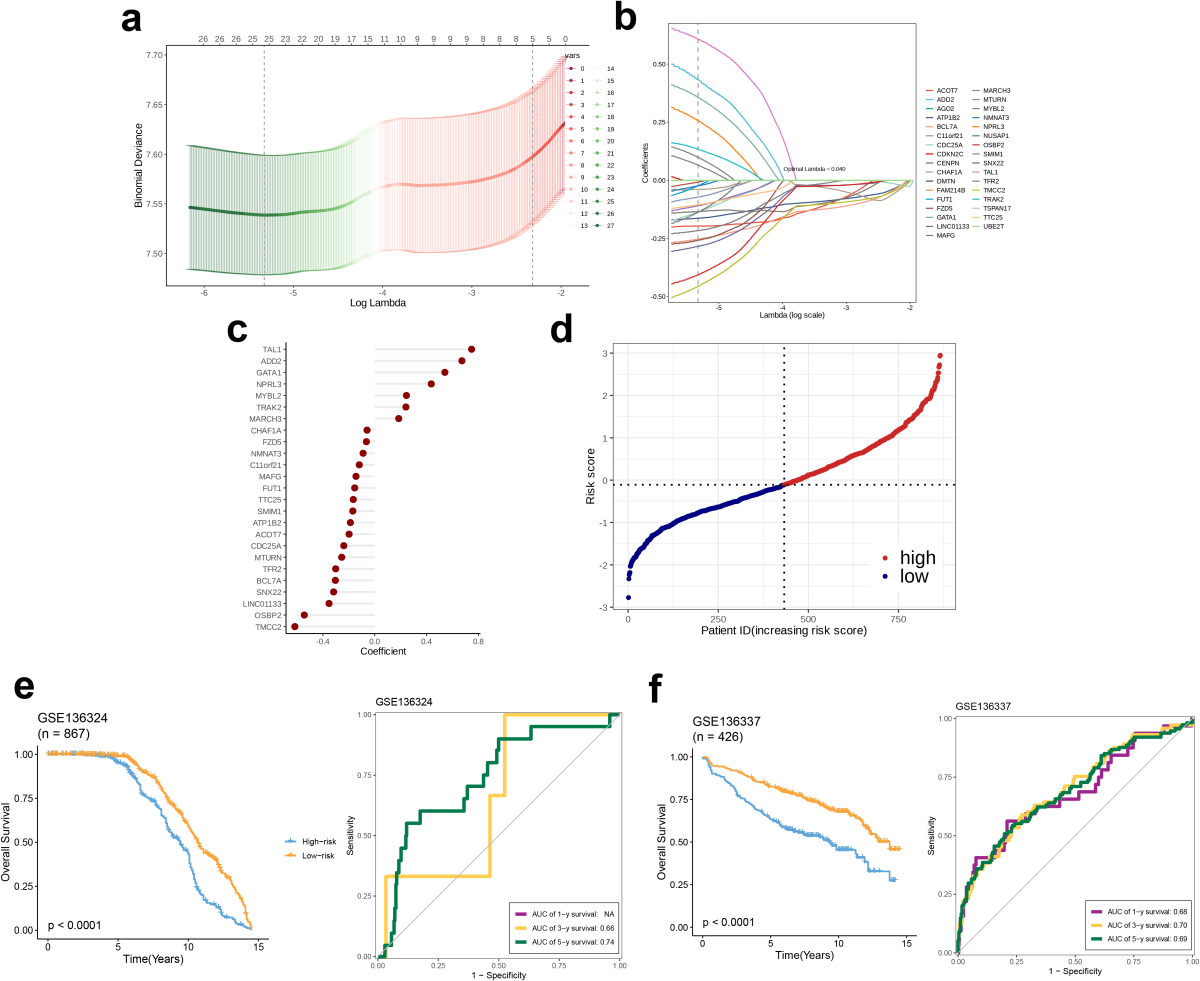
Plasma-related prognostic signature construction and validation. **(a, b)** The selection of prognostic hub genes based on the optimal parameter λ obtained through the Least Absolute Shrinkage and Selection Operator regression analysis. **(c)** A lollipop chart of the coefficients of signature genes determined by the multiCox regression analysis. **(d)** The dotplot demonstrating the risk score of each patient. Survival differences between **(e)** two groups and time-dependent ROC analysis of the model in the GSE136324 and **(f)** two groups and time-dependent ROC analysis of the model in the GSE136337.

LASSO regression analysis performed on the GSE136324 dataset revealed that the mean squared error was minimum when λ = 0.04 (**Figure 6a**). Subsequently, based on the λ value, 25 model genes were selected from the coefficient path distribution plot (**Figure 6b**) and these 25 genes were subjected to multivariate Cox regression analysis, which yielded coefficients for each model gene, thereby enabling the formulation of the prognostic model. Among them, seven genes—TAL1, ADD2, GATA1, NPRL3, MYBL2, TRAK2, and MARCH3—had positive coefficients, while the remaining 18 genes had negative coefficients (**Figure 6c**). Next, using the model, we calculated the risk scores of patients in the GSE136324 dataset and divided them into high- and low-risk groups based on the median score (**Figure 6d**). Subsequently, survival analysis and time-dependent ROC analysis, were performed. KM curve results indicated that although the OS declined over time in high- as well as low-risk groups, the OS of the low-risk group was higher than that in the high-risk group. Analysis conducted using the ROC methodology revealed that the AUCs corresponding to the 3- and 5-year survival rates were 0.66 and 0.74, respectively, thereby indicating a robust capacity of the model for efficacy in the prediction of survival outcomes. (**Figure 6e**). Finally, the model in the independent validation set, GSE136337, using survival analysis and time-dependent ROC analysis. The survival curves of the high- and low-risk groups in GSE136337 and GSE136324 were similar. For the 1-, 3-, and 5-year survival rates, the AUC values obtained were 0.68, 0.70, and 0.69, respectively, strongly validating the model’s robustness and predictive performance (**Figure 6f**).

### 3.7 Biological differences between high and low-risk groups

**Figure 7:**
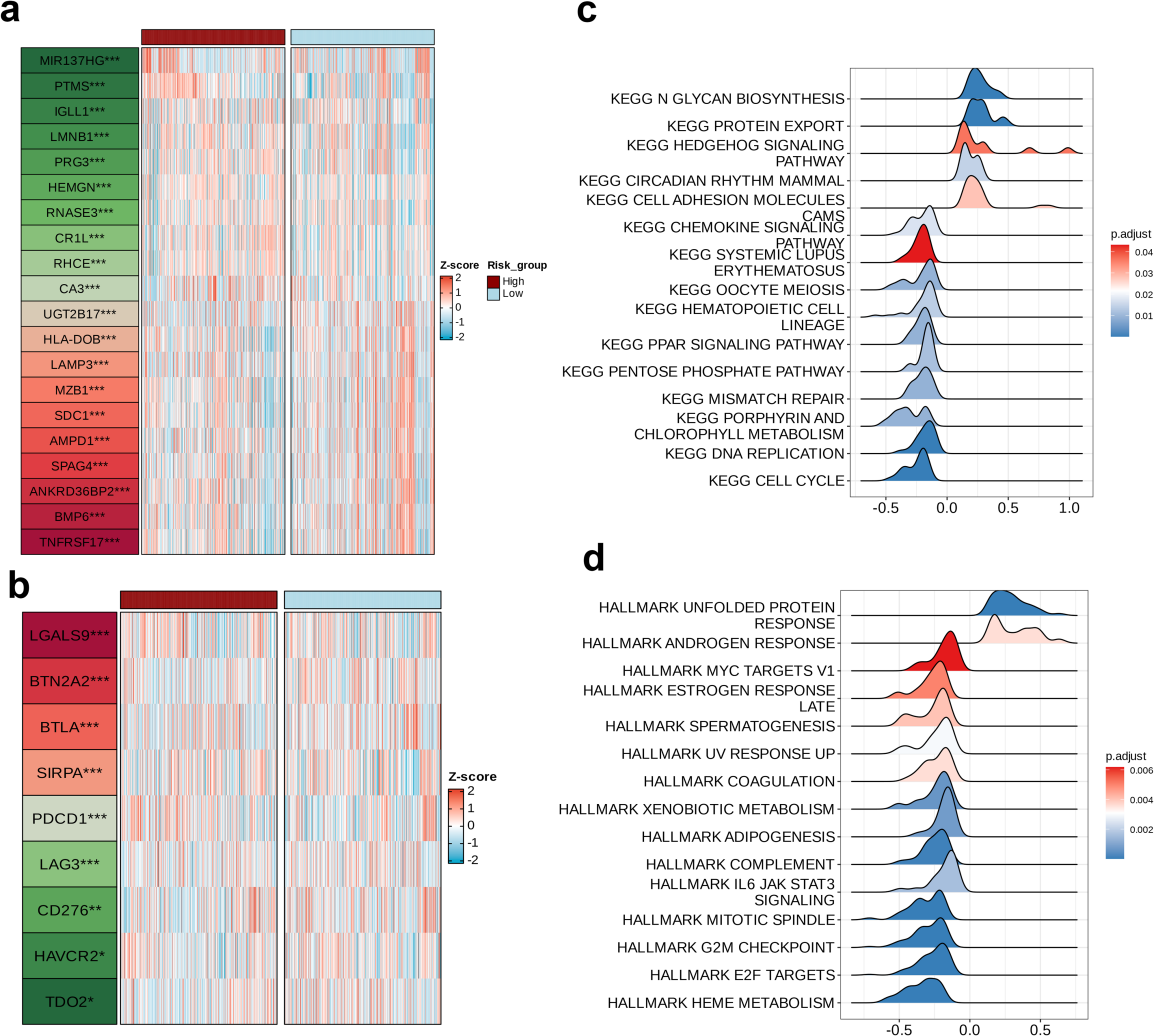
Biological differences among the risk groups. Heatmap showing **(a)** the top 20 dysregulated features between two groups and **(b)** the dysregulated immune checkpoints between two groups. Ridge plots of the **(c)** upregulated Kyoto Encyclopedia of Genes and Genomes pathways in the high-risk group and **(d)** upregulated hallmarks in the high-risk group.

A differential analysis was performed to compare the high and low-risk groups utilizing the ‘limma’ package, identifying the top 20 dysregulated genes and their expression levels in each sample (**Figure 7a**). Subsequently, an in-depth analysis of the expression profiles pertaining to nine immune checkpoints across the two distinct groups were found to be dysregulated, namely LGALS9, BTN2A2, BTLA, SIRPA, PDCD1, LAG3, CD276, HAVCR2, and TDO2 (**Figure 7b**). Next, GSEA was conducted on the high-risk group, initially referencing the KEGG pathways. This revealed that five pathways—N GLYCAN BIOSYNTHESIS, PROTEIN EXPORT, HEDGEHOG SIGNALING PATHWAY, CIRCADIAN RHYTHM MAMMAL, and CELL ADHESION MOLECULES—were enriched in the high-risk group, with N GLYCAN BIOSYNTHESIS showing the most pronounced upregulation. These pathways are involved in various biological processes, including cell development, signaling, interactions, and environmental adaptation, all being essential for maintaining normal cellular and organismal functions (p < 0.05, **Figure 7c**). We subsequently consulted the HALLMARK gene set and discovered that the pathways upregulated in the high-risk cohort, specifically the ANDROGEN RESPONSE and the UNFOLDED PROTEIN RESPONSE, were implicated in the proliferation and survival of tumor cells, with a particularly notable upregulation in the UNFOLDED PROTEIN RESPONSE (p < 0.01, **Figure 7d**).

### 3.8 Association between prognostic models and immunological features

**Figure 8:**
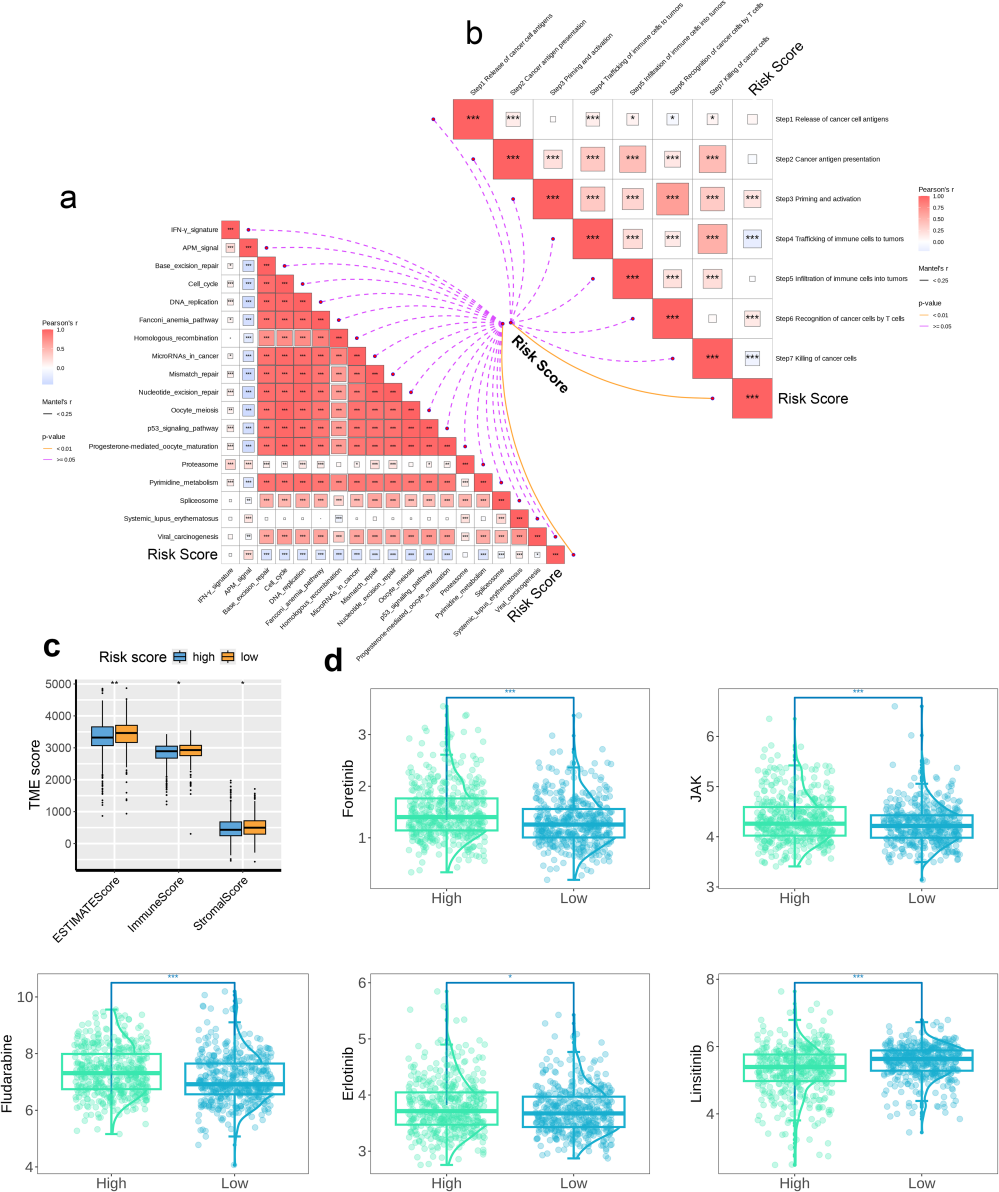
The association between the risk score and immunological features. The correlation between **(a)** the risk score and the immunotherapeutic pathways and **(b)** the risk score and the anti-cancer immunity cycles, **(c)** The TME immune scores determined by the ESTIMATE method between two risk groups and **(d)** The predicted drug sensitivities between two risk groups. * p < 0.05, ** p < 0.01, *** p < 0.001. These significance levels are commonly used in scientific literature to denote the strength of statistical evidence, with more asterisks indicating stronger statistical significance.

**Figure 9:**
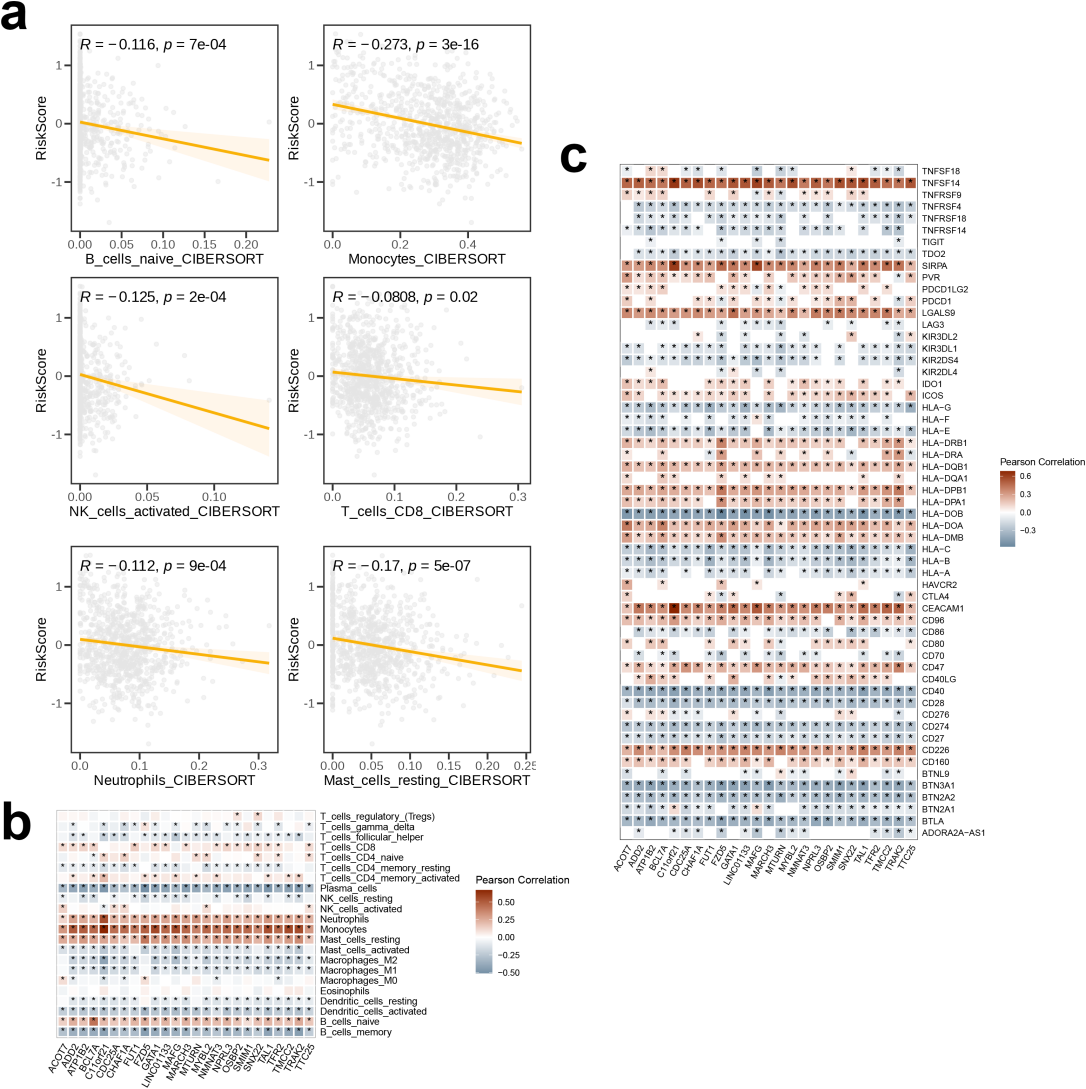
The association among signature genes and immunological features. The correlation **(a)** between risk scores and immune cell infiltration abundances, as estimated by CIBERSORT, **(b)** between signature genes and infiltration levels of 28 immune cell subsets, and **(c)** between signature genes and immune checkpoints. The symbol * indicates statistical significance at the p < 0.05 level. This is the standard threshold for statistical significance in biomedical research, indicating that the observed result would occur by chance less than 5% of the time if there were no actual effect.

The association of 18 immune therapeutic pathways with risk scores was analyzed. The butterfly plot indicated a negative relationship with respect to the majority of immune therapeutic pathways and the risk score, while there exists a positive association between APM_signal and the risk score (p < 0.05, **Figure 8a**). Furthermore, the correlation among the seven sequential stages of the anti-cancer immune cycle, as well as the correlation with the assigned risk scores were assessed. We found positive correlation of steps 3 and 6 with risk scores, while steps 4 and 7 were negatively correlated with risk scores (p < 0.001, **Figure 8b**). Subsequently, comparison of the TME scores between the high- and low-risk groups revealed that within the high-risk cohort, the indices for immune score, stromal score, and ESTIMATE score were reduced compared to the low-risk cohort, suggesting a diminished presence of immune and stromal cell infiltration in the high-risk group (**Figure 8c**). We also calculated the IC50 values of five drugs for the high- and low-risk groups. The insights garnered from the boxplot results demonstrated enhanced sensitivity of the low-risk cohort to Foretinib, JAK inhibitors, Fludarabine, and Erlotinib, whereas the high-risk cohort demonstrated a greater responsiveness to Linsitinib (**Figure 8d**).The relationship between the risk score and the abundance of immune cell infiltration was analyzed using a correlation scatter plot. We observed that six immune cell types—B_cells_naive, Neutrophils, NK_cells_activated, T_cells_CD8, Monocytes, and Mast_cells_resting—were negatively correlated in terms of the risk score, with the strongest correlation being for Monocytes (p < 0.05, **Figure 9a**). Next, we examined the correlation of 25 model genes and the infiltration levels of 22 immune cell subtypes and found that among the immune cell subtypes, Plasma_cells, B_cells_memory, and Dendritic_cells_activated were negatively correlated with all 25 model genes, and the strongest negative correlation was observed for Plasma_cells. In contrast, Neutrophils, Monocytes, Mast_cells_resting, and B_cells_naive were positively correlated with all 25 model genes, and Monocytes showed the strongest positive correlation (p < 0.05, **Figure 9b**). In the final step, heatmap of the analysis of the correlation between 57 immune checkpoints and 25 model genes revealed that most members of the tumor necrosis factor (TNF) superfamily (TNFSF18, TNFSF14, TNFRSF9, TNFRSF4, TNFRSF18, TNFRSF14) were negatively correlated with the model genes; in contrast, TNFSF14 exhibited a strong positive correlation with all 25 model genes. In the programmed cell death protein (PD-1) and its ligand pathway, PDCD1 and PDCD1LG2 showed positive correlation with the model genes, while CD274 was negatively correlated. The Killer Immunoglobulin-like Receptors (KIRs) family (KIR3DL2, KIR3DL1, KIR2DS4, KIR2DL4) and the BTN family (BTNL9, BTN3A1, BTN2A2, BTN2A1) showed negative correlation with the model genes. Among the HLA molecules, HLA-A, HLA-B, HLA-C, HLA-DOB, HLA-E, HLA-F, and HLA-G showed positive correlations with the model genes, while HLA-DRB1, HLA-DRA, HLA-DQB1, HLA-DQA1, HLA-DPB1, HLA-DPA1, HLA-DOA, and HLA-DMB were negatively correlated with the model genes. Among the 57 immune checkpoints, HLA-DOB, CD40, BTN3A1, and BTLA showed the strongest negative correlations with the 25 model genes; in contrast, TNFSF14, SIRPA, LGALS9, CEACAM1, and CD226 exhibited the strongest positive correlations. Among the 25 model genes, C11orf21 demonstrated the strongest positive correlation with the immune checkpoints CEACAM1, TNFSF14, and SIRPA (**Figure 9c**).

## 4 Discussion

MM, the second most common hematologic cancer, has seen an increasing rate of incidence over the years, with high treatment costs. In Australia, with the highest incidence, the average annual treatment cost per patient may reach USD 25,000. Although the direct cause of the disease remain yet to be identified, several risk factors, including advanced age, male gender, African American ethnicity, and MGUS, have been established, most of which are not modifiable. The only potentially changeable risk factor is adult body mass index, although its correlation with MM requires further investigation. Typically, MM progresses from MGUS to SMM, which, despite being asymptomatic, increases the risk of complications such as venous thromboembolism, infections, osteoporosis, and fractures. Patients suffering from MM often experience symptoms such as anemia, bone pain, and fatigue, which severely impact their quality of life. While advancements in treatment have improved the prognosis for patients with MM, relapse remains an inevitable challenge. Emerging therapies have extended survival in patients with relapsed or refractory MM, but their side effects, including cytokine release syndrome and immune effector cell-associated neurotoxicity syndrome, are significant. Furthermore, the effectiveness of approaches like chimeric antigen receptor-T cell therapy and BiTEs is limited by factors such as comorbidities, cytogenetic characteristics, and treatment responsiveness. Therefore, it is essential to study the molecular mechanisms of MM to identify key target genes, enhance disease detection, improve treatment efficacy, and ultimately extend patient survival.

We first performed batch correction and dimensionality reduction clustering on the scRNA-seq data obtained from the TISCH2 database and after clustering and annotation, identified 10 distinct cell subtypes. Focusing on the plasma cell cluster and further dimensionality reduction clustering resulted in two subpopulations, plasma_0 and plasma_1. The plasma_1 subgroup represented a minority of the total population. DEG analysis revealed the top 10 genes with the most significant changes in expression between the two subpopulations. Specifically, IGKC, SNORA31, IGHG2, IGLC1, and EEF1A1P5 were highly expressed in plasma_0, whereas RPS26, RPSA, CCND1, RPL39, and NEAT1 were significantly highly expressed in plasma_1. Subsequently, GO enrichment analysis for the two subpopulations revealed upregulated endoplasmic reticulum-associated protein synthesis and degradation pathways (including the ERAD pathway, Endoplasmic Reticulum to Cytosol Transport, Retrograde Protein Transport, ER to Cytosol, Response to Endoplasmic Reticulum Stress, Protein Exit from Endoplasmic Reticulum, and Ubiquitin-dependent ERAD Pathway) in plasma_0. In contrast, processes related to ribosome-associated protein synthesis and assembly were upregulated in plasma_1 (including Cytoplasmic Translation, Ribosome Biogenesis, Ribosome Assembly, Ribosomal Small Subunit Assembly, Ribosomal Small Subunit Biogenesis, and Ribonucleoprotein Complex Biogenesis). These findings suggest active involvement of plasma cells in protein synthesis, with notable heterogeneity within the plasma cell population.

A cellular communication analysis was conducted between plasma and malignant cells, identifying three distinct cell populations (plasma_0, plasma_1, and malignant) and quantifying the interactions between them. We observed particularly strong interaction between plasma_0 and malignant cells, suggesting that the plasma_0 subset may participate in MM initiation and progression. We then examined the receptor-ligand pairs involved in signaling between the three cell populations, and hypothesized that plasma_0 may influence malignant cells through the MIF-CD74+CXCR4 axis, potentially impacting the progression of MM and potentially serving as a target for disease diagnosis and treatment. Further activity analysis of 29 regulatory factors across the three cell subsets revealed similar activity patterns of most of these regulators in plasma_0 and malignant cells. In addition, the close communication between plasma_0 and malignant cells led us to determine the specificity scores of regulatory factors in plasma_0. Four positive regulators, ranking among the top six in terms of activity, were identified: TCF4(+), FOSL1(+), TAL1(+), and RARG(+). These factors likely play significant regulatory roles in the biological behavior of plasma_0 cells. We assessed the enrichment of 50 pathways using GSVA based on hallmark pathways in the three cell subsets. Notably, six hallmark pathways related to cell proliferation, migration, differentiation, and environmental adaptation—E2F_TARGETS, HEDGEHOG_SIGNALING, ESTROGEN_RESPONSE_EARLY, ESTROGEN_RESPONSE_LATE, and APICAL_SURFACE—were significantly enriched in malignant cells, possibly contributing to tumor progression. In contrast, plasma_0 exhibited enrichment of glycolysis and protein secretion pathways. We hypothesize that plasma_0 cells may influence tumor proliferation, metastasis, immune evasion, and remodeling of TME through the regulation of glycolytic and protein secretion processes. The gene expression signatures of the three cell subsets were then analyzed and 30 genes associated with cancer progression were identified, including TCF7, LEF1, CCR7, LAG3, TIGIT, PDCD1, HAVCR2, CTLA4, ENTPD1, IL2, GZMA, GNLY, PRF1, GZMB, GZMK, IFNG, CD28, TNFRSF14, ICOS, TNFRSF9, ZEB2, HIF1A, ID2, TOX, IKZF2, CCR5, ITGAE, ISG15, TOP2A, and MKI67. Other six genes, including HOPX, TBX21, EOMES, FOXP3, ZNF683, and CD69, were identified as potential tumor suppressors. Our findings suggest that most genes expressing at high levels in the malignant cell population promote cancer progression, whereas the expression of tumor suppressor genes is relatively lower. In plasma_0, both pro-cancer and anti-cancer genes are expressed at lower levels, while in plasma_1, both types of genes are highly expressed. Finally, analysis of DEG analysis between plasma_0 and plasma_1 identified several genes significantly upregulated in plasma_0, potentially serving as potential new biological indicators or drug intervention targets for improving the prognosis of multiple myeloma.

In terms of the top four regulons in plasma_0, we used their corresponding targets as a signature and performed NMF on the training set GSE136324. There two optimal number of clusters, which led to the classification of GSE136324 into clusters C1 and C2. Survival analysis for C1 and C2 Revealed that overall, the prognosis of C1 was better than that of C2. Subsequently, differential expression analysis of metabolism-related genes was carried out for C1 and C2, which focused on genes involved in lipid metabolism, nucleotide metabolism, amino acid metabolism, and glycolysis. Compared to C2, most genes in the amino acid metabolism pathway were expressed at higher levels in C1, while the other three pathways showed higher activation in C2. We speculate that the activation of classic metabolic pathways provides strong support for tumor progression, contributing to poorer prognosis. GSEA was performed on C1 and C2; compared to C1, immune response-related pathways (such as Phagosome, Chemokine signaling pathway, Neutrophil extracellular trap formation) and cell cycle-related processes (such as Mismatch repair, Cell cycle, DNA replication) were upregulated in C2. Meanwhile, pathways related to protein synthesis, modification, and secretion (including N-Glycan biosynthesis, various types of N-glycan biosynthesis, Protein export, Ribosome biogenesis in eukaryotes, other types of O-glycan biosynthesis) and cell signaling pathways (such as the Hippo signaling pathway and ECM-receptor interaction) were downregulated in C2. While some immune responses were enhanced in C2, tumor cells may escape immune surveillance through immune evasion mechanisms, and disbalanced cell cycle may result in more aggressive tumor behavior. Furthermore, the downregulation of protein synthesis, modification, secretion-related pathways, and cell signaling pathways could impair the survival and environmental adaptability of the cell. We suggest that these factors may collectively contribute to the poor prognosis of the C2 group.

Subsequently, WGCNA was conducted to construct a co-expression network based on the optimal soft threshold. We found the strongest positive correlation of the Module_blue with the Cluster, and therefore, hub genes were extracted from this module. Subsequently, a GO enrichment analysis on the hub genes Revealed that the most significantly enriched terms were those associated with cell division, such as chromosome segregation, tubulin binding, and spindle formation. This finding suggests potential involvement of the hub genes in regulating tumor cell proliferation and migration, potentially linking them to the malignancy and prognosis of the tumor.

Subsequently, a prognostic model was established empoying multiCox regression analyses and LASSO. A λ value of 25 was selected, allowing us to identify 25 genes (TAL1, ADD2, GATA1, NPRL3, MYBL2, TRAK2, MARCH3, CHAF1A, FZD5, NMNAT3, C11orf21, MAFG, FUT1, TTC25, SMIM1, ATP1B2, ACOT7, CDC25A, MTURN, TFR2, BCL7A, SNX22, LINCO1133, OSBP2, TMCC2). TAL1 (also known as SCL), a basic helix-loop-helix (bHLH) transcription factor, plays a critical role in hematopoiesis (32), expressed predominantly in the adult vascular and hematopoietic systems, especially in hematopoietic stem cells, erythroblasts, megakaryocytes, and mast cell progenitors (33). The Adducin protein family, comprising α, β, and γ subtypes, includes ADD2, which encodes the Adducin β protein and is expressed mainly in the hematopoietic system and brain tissues (34). GATA1, a GATA transcription factor family member, is predominantly expressed in erythrocytes, megakaryocytes, eosinophils, mast cells, and DCs (35). This protein can recognize GATA sequences and promote the differentiation of erythrocytes and megakaryocytes by regulating target genes (36). Nitrogen permease regulator-like 3 (NPRL3), together with NPRL2 and pleckstrin domain-containing 5, forms a GTPase-activating protein (GAP) complex known as GATOR1 (37), which regulates mTORC1 signaling and subsequently influences cortical development. NPRL3 mutations have been associated with familial and sporadic focal cortical dysplasia type IIa (38). The MYBL2 gene, also called B-MYB, a member of the transcription factor family, is involved in myeloproliferative disorders, playing a key role in the proliferation, differentiation, and cell cycle regulation of proliferating cells. MYBL2 overexpression has been observed in various cancers, including acute myeloid leukemia, hepatocellular carcinoma, and breast cancer; overexpression in colorectal cancer is thought to correlate with poor prognosis (39). Trafficking protein, kinesin binding 2 (TRAK2), a member of the TRAK family of proteins, acts as a motility linker protein by binding to Miro1/2, thus anchoring mitochondria to motor proteins (40, 41). E3 ubiquitin ligases are the membrane-associated RING-CH-type finger (MARCH) proteins. The MARCH family comprises four subgroups: MARCH1 and MARCH8, MARCH2 and MARCH3, MARCH7 and MARCH10, and MARCH4, MARCH9, and MARCH11 (42). Among these, MARCH3 regulates the interleukin (IL)-3-induced inflammatory response in the opposite direction by mediating K48-linked polyubiquitination and the degradation of IL-3 receptor alpha (IL-3Rα), thereby carrying out its corresponding immune function (43). The transmembrane and coiled-coil domain containing 2 (TMCC2) possibly has a role in maintaining erythropoiesis in mice (44), and may be associated with the risk of Alzheimer’s disease (45). Oxysterol-binding protein 2 (OSBP2), an OSBP family member (46), is specifically expressed in the retina, pineal gland, testes, and fetal liver. Studies suggest that OSBP2 is possibly involved in regulating apoptosis during development (47). LINC01133, a long non-coding RNA, has been shown to contribute to poor prognosis in lung squamous cell carcinoma and osteosarcoma (48), though its role in MM remains unclear. The function of SNX22 remains largely unstudied. A member of the BCL7 family, B-cell lymphoma 7 protein family member A (BCL7A), is believed to exert a tumor-suppressive effect in gliomas, colorectal cancer, and ovarian cancer, and acts as a biomarker (49), though its role in MM has been rarely investigated. Transferrin receptor 2 (TFR2) is mainly expressed in the liver and erythrocytes; although its precise role in iron metabolism is not fully understood, it is potentially crucial for maintaining iron homeostasis (50). To date, no studies have been published on the MTURN gene. CDC25A, a member of the cell division cycle 25 (CDC25) phosphatase family, activates cyclin-dependent kinases through the dephosphorylation of threonine and tyrosine residues, thus regulating the cell cycle (51). CDC25A overexpression in tumors has been associated with poor prognosis (52). Acyl-CoA thioesterases (Acots), including types I and II, play roles in lipid metabolism and other cellular processes dependent on this pathway by catalyzing the hydrolysis of fatty acyl-CoA ester molecules (53). ACOT7, the most widely studied type II Acot, is highly expressed in brain tissues and has neuroprotective effects (54). ATP1B2, a plasma membrane pump, is broadly expressed in brain tissues and may be associated with Parkinson’s disease (PD) (55). Nonetheless, research on most of these genes in the context of MM is still insufficient. Subsequently, we performed multiCox regression analysis and calculated the coefficients for these 25 genes. Employing the median risk score, we stratified the samples in the GSE136324 dataset into high- and low-risk cohorts. Survival analysis revealed better prognosis for the low-risk group than the high-risk group, and the model demonstrated good predictive performance based on the ROC analysis. Further validation of the model’s efficacy in the GSE136337 dataset confirmed its strong generalizability.

A differential analysis between high- and low-risk groups identified the 20 genes with the highest differential expression, and the differential expression of 9 immune checkpoint-related genes was also investigated. A GSEA on the high-risk group revealed four significantly upregulated KEGG pathways, including protein modification and secretion-related pathways (N-Glycan Biosynthesis, Protein Export), the developmental signaling pathway (Hedgehog Signaling Pathway), and the cell adhesion and migration-related pathway (Cell Adhesion Molecules). The Hedgehog signaling pathway is a critical cellular signaling pathway that participates in various biological processes, including cell proliferation, differentiation, and stem cell maintenance. We hypothesize that the model genes possibly enhance tumor proliferation, migration, and survival by upregulating these pathways, thereby leading to poor prognosis, suggesting potential intervention points for targeted therapy. We identified two significantly upregulated Hallmark pathways in the high-risk group: Hallmark Unfolded Protein Response and Hallmark Androgen Response. Activation of these pathways can promote tumor cell proliferation and survival, and contribute to the poor prognosis in MM.

We then assessed the correlation between risk scores and 18 immune therapy pathways, and found that most of the effective immune therapy pathways were associated with lower risk scores, suggesting the sensitivity of MM to these treatments. Next, we examined the relationship between the seven steps of the anti-cancer immune cycle and risk scores and found a significantly negative correlation of Step 4 Trafficking of immune cells to tumors and Step 7 Killing of cancer cells with the risk score, indicating that these two steps contribute to better treatment outcomes for MM. Conversely, Step 3 Priming and activation, as well as Step 6 Recognition of cancer cells by T cells were positively correlated with the risk score, suggesting that adjustments to these steps may be required to improve treatment efficacy. Subsequently, the TME scores for high- and low-risk groups revealed that the proportion of immune and stromal cells was higher in the low-risk group compared to that in the high-risk group. This observation suggests that the high-risk group may be in a relatively immune-suppressive state, potentially facilitating immune evasion by tumor cells and promoting tumor progression. In contrast, the immune response in the low-risk group appeared to be more active, conferring a stronger anti-tumor property. We then compared the IC50 values of five drugs—Foretinib, JAK inhibitors, Fludarabine, Linsitinib, and Erlotinib—in the high- and low-risk groups, and found that the drug sensitivity in the high-risk group was significantly lower than that in the low-risk group. Specifically, the low-risk group exhibited greater sensitivity to Foretinib, JAK inhibitors, Fludarabine, and Erlotinib, while, the high-risk cohort demonstrated enhanced sensitivity to Linsitinib. Subsequently, examination of the relationship between risk scores and the infiltration levels of six distinct immune cell types, revealed a consistent negative correlation across all cell types. This suggests that a higher degree of immune cell infiltration may help in inhibiting the progression of MM. Among the related immune reactions, B cells naive, monocytes, activated NK cells, CD8+ T cells, neutrophils, and resting mast cells exhibited critical roles in combating MM. Additionally, assessment of the correlation between 25 model genes and the distribution of 22 immune cell subsets within the tissue Showed a negative correlation in association with the 25 model genes and plasma cells, memory B cells, as well as activated dendritic cells, while neutrophils, monocytes, resting mast cells, and naive B cells were positively correlated with these genes.

Finally, we examined the correlation between 57 immune checkpoints and the 25 model genes, and found that TNFSF14 was being highly correlated with the model genes for MM prognosis. This suggests that TNFSF14 may play a key role in MM pathogenesis and progression and could potentially serve as a target for the treatment of MM.

## 5 Conclusion

Single-cell sequencing analysis was conducted with a focus on the role of plasma cells in MM and their potential underlying mechanisms. Gene expression profiles and pathway enrichment differences among different plasma cell subtypes were compared, while also integrating malignant subpopulations for analyzing cell communication and transcriptional regulation. Subsequently, NMF was applied for clustering and inter-group survival differences, and gene variations related to metabolic pathways were analyzed. Next, hub genes were extracted through WCGNA and a prognostic prediction model using LASSO and multi-variable Cox regression was constructed. Finally, the correlation was explored between the risk model and tumor immune therapy, the TME, as well as immune checkpoints. These findings offer new research directions for improved treatment strategies and patient prognosis in MM.

## Data Availability

The original contributions presented in the study are included in the article/supplementary material. Further inquiries can be directed to the corresponding author.
